# The Influence of Agroecological Intensification on Dominant and Rare Microbial Communities Across Diverse European Countries

**DOI:** 10.1007/s00248-025-02655-5

**Published:** 2025-12-19

**Authors:** Ángel Carrascosa-Robles, Jose Antonio Pascual, Alessandra Trinchera, Elena Testani, Sébastien Fontaine, Sara Sanchez-Moreno, Skaidrė Supronienė, Simon Sail, Jim Rasmussen, Marjoleine Hanegraaf, Margarita Ros

**Affiliations:** 1 Department of Soil and Water Conservation and Organic Waste Management, Centro de Edafologia y Biología Aplicada del Segura (CEBAS- CSIC), Murcia, 30100 Spain; 2 Council for Agricultural Research and Economics (CREA), Rome, 00184 Italy; 3 French National Institute for Agriculture, Food, and Environment (INRAE), VetAgroSup, University Clermont Auvergne, UMR Ecosystème Prairial, Clermont-Ferrand, 63100 France; 4 Spanish National Institute for Agricultural and Food Research and Technology, Spanish National Re-search Council (INIA-CSIC), Madrid, 28040 Spain; 5 Lithuanian Research Centre for Agriculture and Forestry (LAMMC), Institute of Agriculture, Akademija, 58344 Lithuania; 6 Department of Sustainability, Systems & Prospectives-Unit of Soil, Water and Integrated Crop Production, The Walloon Agricultural Research Centre (CRAW), Gembloux, 5030 Belgium; 7 Department of Agroecology (AU-AGRO), Aarhus University, Viborg, 8000 Denmark; 8 Wageningen Plant Research, Wageningen University & Research, Wageningen, 6700 The Netherlands

**Keywords:** Microbial sub-communities, 16SrRNA genes, ITS genes, Sustainability-promoting agricultural practices

## Abstract

Intensive land use leads to the degradation of agroecosystems, resulting in long-term losses in agricultural productivity. In contrast, sustainable management is known to improve soil fertility directly and indirectly through changes in the soil microbiota, which plays a fundamental role in agroecosystems by influencing nutrient biogeochemical processes and through symbiotic relationships with crops. In this study, we used amplicon sequencing to investigate changes occurring in dominant and rare sub-communities of bacteria and fungi in agricultural soils from seven European countries under different number of agroecological cropping systems: one and two sustainability-promoting practices or none. Both sub-communities were structured along a latitude gradient, reflecting bioclimatic differences across Europe, especially the fungal communities. Differences in the bacterial and fungal sub-communities’ structure were greater under the 2SP treatment than under 1SP, particularly within the fungal dominant sub-community, which changed by sustainability-promoting practices across more sites. In both fungal and bacterial communities, we identified specific taxa associated with carbon and nutrient cycling, pathogen suppression, or plant growth promotion.

**Supplementary Information:**

The online version contains supplementary material available at 10.1007/s00248-025-02655-5.

## Introduction

Soil biota is essential in providing ecosystem services [1] thanks to the key role of soil microorganisms in diverse soil processes such as soil nutrient cycling, organic matter decomposition, C stock regulation, plant productivity, and pest control [2]. Land-use perturbation has been identified as one of the main anthropic pressures affecting soil microbial diversity and resulting in community composition shifts. This should be considered when planning soil management [3, 4]. Conventional management, focused on agricultural productivity, leads to large-scale soil degradation, reducing soil health and fertility among other negative environmental impacts [5–7]. The greatest challenge in agriculture is to increase crop production, while reducing negative environmental impacts by implementing sustainable solutions in agriculture. Agricultural practices that are permanent and compatible with the environment are of great importance to soil and natural resource sustainability in the long term. Therefore, determining the most economical and fastest way to implement these practices in agriculture should be a priority for farmers [8, 9]. According to Mehmet Tuğrul [5] sustainability-promoting agricultural practices are mainly focus on increasing soil productivity and reducing the harmful effects of agricultural practices on the climate, soil, water, the environment, and human health. This type of agriculture reduces the use of non-renewable sources and employs renewable resources to improve agricultural production [5, 10, 11].

Sustainable agriculture comprises the implementation of alternative agricultural practices designed to promote sustainability, enhance soil health, preserve biodiversity, and maintain productivity while minimising environmental impacts [12]. These sustainability-promoting practices include agroforestry, intercropping, crop rotation, green manuring, conservation tillage, cover crops, and adopting biofertilisers [13]. Crop rotation can help conserve, maintain, and replenish soil resources, including organic matter, nutrient inputs, and physical and chemical properties and it has an important influence on the soil’s microbial properties [14]. The appropriate choice and sequence of crops in rotation are crucial to optimize nutrient cycling within the field system and minimize losses over the short and long term [15, 16]. No-tillage practices lead to higher C and N concentrations and water content in the soil. Microbial population size and diversity in agricultural soils can be affected not only by individual practices such as tillage [17, 18] and crop residue retention [19], but also, by their combined effects [20]. Green manuring is the practice of incorporating undecomposed green plants from the same field or another into the soil to maintain the nutrient supply for the next crop [21]. Green-manured crops promote microbial growth and their activity in the soil by releasing nutrients and energy-supplying materials, such as root exudates, eventually enhancing soil fertility and health [22]. However, other studies have reported that the effects of diversification or intensification of sustainability-promoting practices may vary between dominant and rare sub-communities, or may even be less pronounced than those driven by other factors such as climate conditions or intrinsic soil properties [23, 24]. Furthermore, promoting truly sustainable agricultural systems relies on the simultaneous application and study of multiple sustainability-promoting practices, as individual practices alone are often insufficient to achieve long-term sustainability goals [25]. This highlights the need to disentangle how sustainability-promoting practices, and their number, interact with environmental and edaphic drivers to shape microbial community composition [23, 26, 27].

Understanding the potential of the microbiome in agriculture leads us to use it as an inoculant or to select more efficient microbial groups for plant development [28]. This could reduce the incidence of plant disease [9], increase agricultural production, and decrease chemical inputs [29], thereby contributing to more sustainable agriculture. Soil microbial communities are highly diverse and contain dominant and rare taxa that play complementary roles in regulating multiple soil processes and maintaining ecosystem functions [30]: dominant taxa channel the most of the energy and biomass through ecosystems, ensuring its stability and maintenance of soil agroecosystems [31, 32]; whereas rare taxa maintain ecosystem function under environmental conditions changes [33, 34], exhibiting greater sensitivity to environmental factors than common species [8, 35] and acting as reservoirs of genetic diversity and supporting specific ecosystem functional traits [36, 37]. Taking together, these complementary roles highlight the ecological significance of the soil microbial community’s distribution, and, studying those sub-communities under different levels of agricultural intensification is therefore of particular interest, as management practices may differentially shape these groups and therefore, influence the capacity of soils to sustain fertility, productivity, and resilience [27, 38]. However, the processes shaping rare bacterial species remain largely unknown or have been frequently overlooked [39–41], therefore, a deeper understanding of the ecological attributes of common versus rare soil microbial communities could enhance our ability to predict how soils ecosystems responds to environmental change and to identify which taxa should be protected. Although taxonomic approaches do not directly measure microbial functions, they provide valuable information of soil functionality, as shifts in the abundance of particular microbial groups are often associated with changes in key ecological processes [42, 43]. Consequently, we can evaluate whether protecting only common taxa would be enough to safeguard soil ecosystem services [44].

In this work, we aimed to analyse the soil bacterial and fungal communities through the amplicon sequencing analysis of soil DNA from long term experiments under a different number of sustainability-promoting agronomical practices across 7 European countries, in order to (i) assess the European-scale effects of the number of sustainability-promoting practices on dominant and rare microbial sub-communities; (ii) explore taxonomic changes of microbial sub-communities at the phylum and genus levels; and (iii) evaluate the influence of edaphoclimatic conditions on the microbial sub-community structure. Our hypothesis is that increasing the number of sustainability-promoting agricultural practices influences dominant communities and rare microbial sub-communities differently, due changes in soil properties, such as organic matter and soil nutrients content. By simultaneously considering dominant and rare microbial taxa across multiple long-term field experiments and diverse European environments, this study addresses a current knowledge gap on how the intensity of sustainability-promoting practices shapes soil microbial communities at a continental scale, thereby providing novel insights into the microbial shifts underpinning sustainable agriculture.

## Materials and Methods

### Experiment Design and Sampling

This study is part of the EJP Soil Project (AGROECOSeqC). It was conducted across various European countries: Spain (SPA), Lithuania (LIT), the Netherlands (NET), Belgium (BEL), Denmark (DEN), France (FRA), and Italy (ITA), representing a range of edaphoclimatic conditions (Fig. S1). In each country hereafter referred to as core sites (CSs), we selected long-term experiments with different agricultural practices: increasing crop diversity (cover crops, intercropping, and rotation), reducing soil disturbance (tillage reduction) and the use of organic inputs (plant residue, manure and compost). These treatments were designed as a gradient of number of sustainability-promoting agricultural practices: (i) absence of sustainability-promoting practices (0SP); (ii) implementation of one sustainability-promoting practice (1SP) and (iii) implementation of two sustainability-promoting practices (2SP), according to previous studies [26, 45]. The specific practices within each site across different core sites are in Table 1, more information concerning the experimental design of each core site can be found in Doyeni et al. [46].

**Table 1:** Location and description of the diversification strategies in the experiments

**Location**	**Site**	**Establishment Date**	**Sampling** **Date**	**Plot Size**	**Main Crop**	**Sustainability-Promoting Practices**	**Practices**
Spain, Alcalá de Henares	SPA	1994			Wheat	0SP	Monocrop, tillage
			May 2023	250 m^2^		1SP	Rotation, tillage
						2SP	Rotation, no tillage
France, Clermont	FRA	2016			Wheat	0SP	Monocrop, tillage
			May 2023	490.9 cm^2^		1SP	Wheat and legumes
						2SP	Wheat, legumes and cover crops
Italy, Rome	ITA	2017			Organic Apricot	0SP	Organic fertilizer (compost) monocrop, tillage
			April 2023	132 m^2^		1SP	Compost, mixed cover crops and tillage
						2SP	Compost, spontaneous cover and no tillage
Belgium, Gembloux	BEL	1959			Sugar beet, winter wheat, winter barley	0SP	Residue´s export, no cattle manure
			April 2023	48 m^2^		1SP	Residue´s export, cattle manure
						2SP	Residues applied as green manure, no cattle manure, cover crops
Netherlands, Wageningen	NET	2016			Cereals	0SP	Fallow
			April 2023	50 m^2^		1SP	Vetch and oats cover
						2SP	Vetch, oat and radish cover
Lithuania, Akademija	LIT	2013			Cereals	0SP	Tillage, no cover crop
			October 2022	45 m^2^		1SP	No tillage, no cover crop
						2SP	No tillage, cover crop
Denmark, Foulum	DEN	2021			Ryegrass	0SP	Monocrop
			July 2022	18 m^2^		1SP	Six species mixtures of rygrass
						2SP	Ryegrass and white clover

Samples were collected at peak of green biomass corresponding to the period of maximum nutrient uptake for each core site. The experiments followed a completely randomized block design. Four blocks with 3 treatments (plots), a total of 12 plots per CS. From each plot, four sub-samples were collected from the soil surface layer (0–20 cm depth range), covering approximately a 5 cm thick by 15 cm wide and then pooled together to form one composite sample. Soil samples were sieved using a 2 mm mesh. For physicochemical analyses, the samples were air dried and stored at 4 °C; for molecular analyses, fresh samples were stored at −20 °C according to [14, 47].

### Soil Physical, Physicochemical, and Chemical Properties

The total nitrogen (TN), total carbon (TC) and total organic carbon (TOC) were determined using an elemental CHNS-O analyser (Truspec CN, Leco, St. Joseph, Mich., USA). The nutrients were measured using ICP-MS (7500CE, Agilent, Santa Clara, CA, USA). The available P was measured following Korndorfer et al. [48]. The nitrates and ammonium were measured following the Italian Gazette no. 248 of 21/10/1999 “Official Methods for Soil Chemical Analysis” Method 19, 1999.

### Soil DNA Extraction and Sequencing

The DNA was extracted with the DNeasy PowerSoil Pro kit (Quiagen, Germany) from 0.5 g of soil. The DNA was purified with the QIAquick Gel kit (Qiagen). To measure the quality of the DNA, electrophoresis was performed on a 1.5% agarose gel. In addition, a NanoDrop 2000 fluorospectrometer (Thermo Fisher Scientific, Waltham, MA, USA) was used to quantify the DNA extraction yield. To avoid the excessive co-amplification of plasmids, chloroplast and mitochondrial rRNA gene sequences, the resulting 16S and ITS amplicons were tagged to Peptide Nucleic Acid (PNA) clamps in the PCR, avoiding the amplification of this non-targeted DNA [49, 50]. The V3-V4 region of bacterial 16S rRNA was amplified using the primer pair 341 F (5′-CCTACGGGNBGCASCAG-3′) and 806R (5´-GACTACNVGGGTATCTAATCC-3′) [51]. The ITS2 region of fungi was amplified using the primer pair ITS2 – fiTS7 (5’-GTGARTCATCGAATCTTTG-3’) and ITS4 (5’-TCCTCCGCTTATTGATATGC-3’) [52]. DNA sequencing was performed at the Instituto de Parasitología y Biomedicina “Lopez-Neyra” (CSIC, Spain) with Illumina MiSeq technology (Illumina Inc., CA, USA) using a paired 2 × 300 bp (PE 300) strategy. The libraries were constructed with the Nextera XT v2 DNA Library Preparation kit (Illumina Inc).

### Sequencing Data Processing

The demultiplexed sequence quality was tested using the FASTQC program v 0.12.1 [53]. The raw sequences were trimmed, denoised, merged, and checked for chimeras, and the singletons were removed using the DADA2 v 1.22 pipeline [54] on R v 4.1.2 [55] for Rocky Linux. For bacterial sequences, we trimmed the first 19 and 21 nucleotides of the sequences, while for fungal sequences, primers were removed using Cutadapt v 4.9 as the internal transcribed spacer (ITS) region exhibits high length variability across taxa, making fixed-length trimming unsuitable [56]. The bacterial and fungal sequences were trimmed using a quality score threshold of five and two, respectively. No further trimming was carried out. The amplicon sequence variant (ASV) taxonomy assignment was performed using the SILVA v 138.1 [57] database for bacteria and the UNITE v 9.0 database for fungi [58]. ASVs that were not assigned to a known phylum, as well as singletons, were removed from the dataset. To facilitate the comparison among samples, the ASV tables were rarefied to the lowest sequencing depth observed across all samples. After processing, the bacterial and fungal datasets yielded 21,704 and 9,071 reads, respectively.

### Climate Data Collection

The climatic data was obtained from the WorldClim database [59], using the data from 1970 to 2000 at a resolution of 30 s (~ 1 km^2^). We extracted the following data: precipitation, annual minimum temperature, and annual maximum temperature using the function *rast* from the “Terra” package [60]. The bioclimatic variables were obtained using the *biovars* function from the “Dismo” package [61].

### Statistical Analysis

All statistical analyses were conducted using R v 4.1.2 [55]. The sample data, ASV reads, and taxonomic assignation were handled using microeco objects from the “microeco” package [62]. Distinguishing between dominant and rare sub-communities is of great relevance, as microbial taxa typically follow a power-law distribution, where a few taxa are dominant and comprise the majority of individuals in the community, whereas the majority of taxa are considered rare. However, determining the boundary is not obvious. Therefore, different thresholds have been proposed in the literature, such as 0.1% or 0.01% or even including an intermediate group to distinguish dominant, intermediate, and rare taxa [63]. In our study, ASVs with a relative abundance greater than 0.1% in each sample were classified as dominant, whereas those below 0.1% were considered rare, according to previous studies [8, 32]. The analyses were performed separately for the dominant and rare subsets of both bacterial and fungal communities.

The alpha diversity indices, Shannon and Richness, were calculated using the function *cal_alphadiv* from the “microeco” package. The community’s structure was visualized in the “microeco” package using Principal Coordinate Analysis (PCoA) based on the Bray-Curtis dissimilarity matrix, and quantified with a permutational multivariate analysis of variance (PERMANOVA) using the *adonis2* function from the “vegan” package [64]. To study the effects of the geographic position and agricultural systems, we first performed a two-way PERMANOVA with 999 permutations for each community. Since the interaction (site x treatment) was significant, we conducted a one-way PERMANOVA with 999 permutations to evaluate the effects of the treatments within each core site. Differences across treatments were calculated according to a Duncan´s Multiple Range post hoc test for PCoA components 1 and 2.

To study the effect size of the number of sustainability-promoting agricultural practices on the alpha diversity indices and the abundances of microbial phyla and genera, we calculated the natural log of response ratios and their confidence intervals at a 95% confidence level using a modified version of the function *logRespRatio* from the “ARPobservation” package [65]. To avoid zero values in the databases, we added 10^− 4^ to all the values, and to test whether the effect size changes were significant, a lineal mixed model (LMM) were fitted, using the *lmer* function from the “lme4” package [66] using the site as random factor, avoiding the inherent differences to each core site, due to the different practices applied or edaphoclimatic conditions. The effect size of phylum taxa was plotted using a forest plot, whereas the genus taxa were plotted using a volcano plot. A principal component analysis (PCA) was performed for the bioclimatic variables obtained from the WorldClim data, and the soil properties (total carbon, organic carbon, total nitrogen, NH_4_^+^, NO_3_, and available phosphorous) using the “Factoextra” and “FactoMineR” packages [67, 68]. To summarize the main patterns in the microbial composition and facilitate the interpretation of their relationships with environmental variables, we conducted a Spearman´s correlation test using the *cor.test* function from the “stats” R package [55] followed by a linear regression analysis using the *lmperm* function from the “Permuco” package [69] between the geographic coordinates (latitude and longitude) and the first components (PCA1) from the climatic data and soil properties PCA analysis with the dominant/rare bacterial and fungal communities’ composition using the first component of the PCoA analysis (PCoA1) based on the Bray-Curtis dissimilarity matrix. Partial Mantel tests were used to determine Pearson´s correlations between individual environmental variables and their Euclidean distance matrix with the sub-communities Bray – Curtis dissimilarity, controlling for the effect of geographical distance as cofounding variable and 999 permutations using the *mantel.test* function from the “vegan” package.

## Results

### Relative Abundance and Percentage of Soil Bacteria and Fungi Sub-Communities

**Fig. 1:**
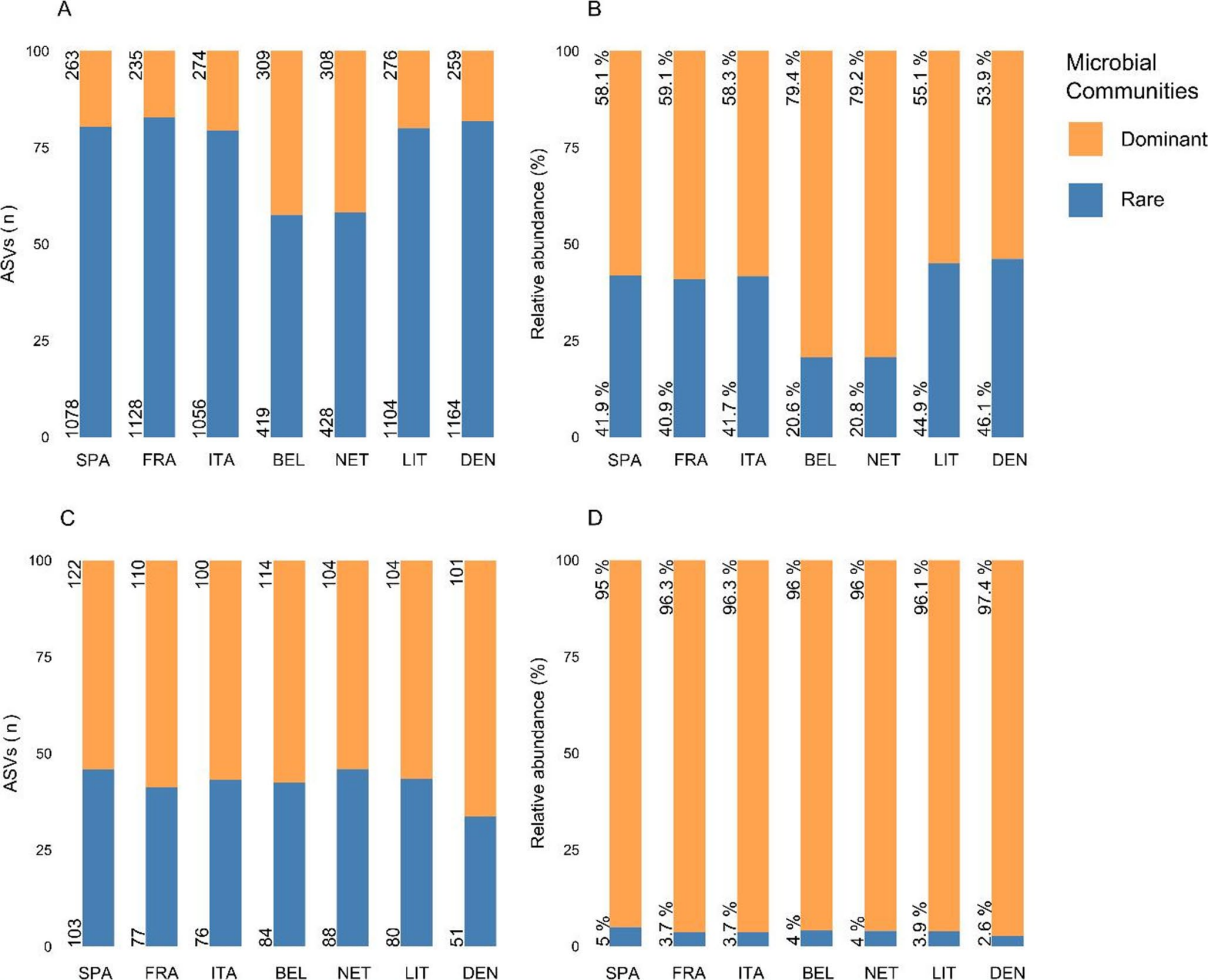
Number of total ASVs corresponding to the bacterial (**A**) and fungal (**C**) sub-communities in each core site and the relative abundance of those ASVs in the whole bacterial (**B**) and fungal (**D**) community

The number of ASVs classified as dominant or rare in the bacterial and fungal sub-communities along with their relative abundance in the community varied slightly among the core sites (Fig. 1). In most of the core sites, approximately 75% of the bacterial ASVs were classified as rare, contributing to 45% of the total relative abundance (Fig. 1A - B), whereas the dominant bacteria showed approximately 25% of the ASVs, with a relative abundance of 55%. However, for the core sites in BEL and NET, the dominant bacteria comprised around 60% of ASVs, accounting for 20% of the total relative abundance (Fig. 1A-B).

The classification of the dominant and rare fungi was more consistent across the core sites (Fig. 1C-D). On average, 41% of the ASVs corresponded to rare fungal communities, and they contributed, on an average, 4% of the relative abundance of the fungal community, while dominant fungi showed 59% of the ASVs and 96% of abundance (Fig. 1C-D).

### Effect of Sustainability-Promoting Practices on the Diversity Indices of Soil Bacteria and Fungi Sub-Communities

**Fig. 2:**
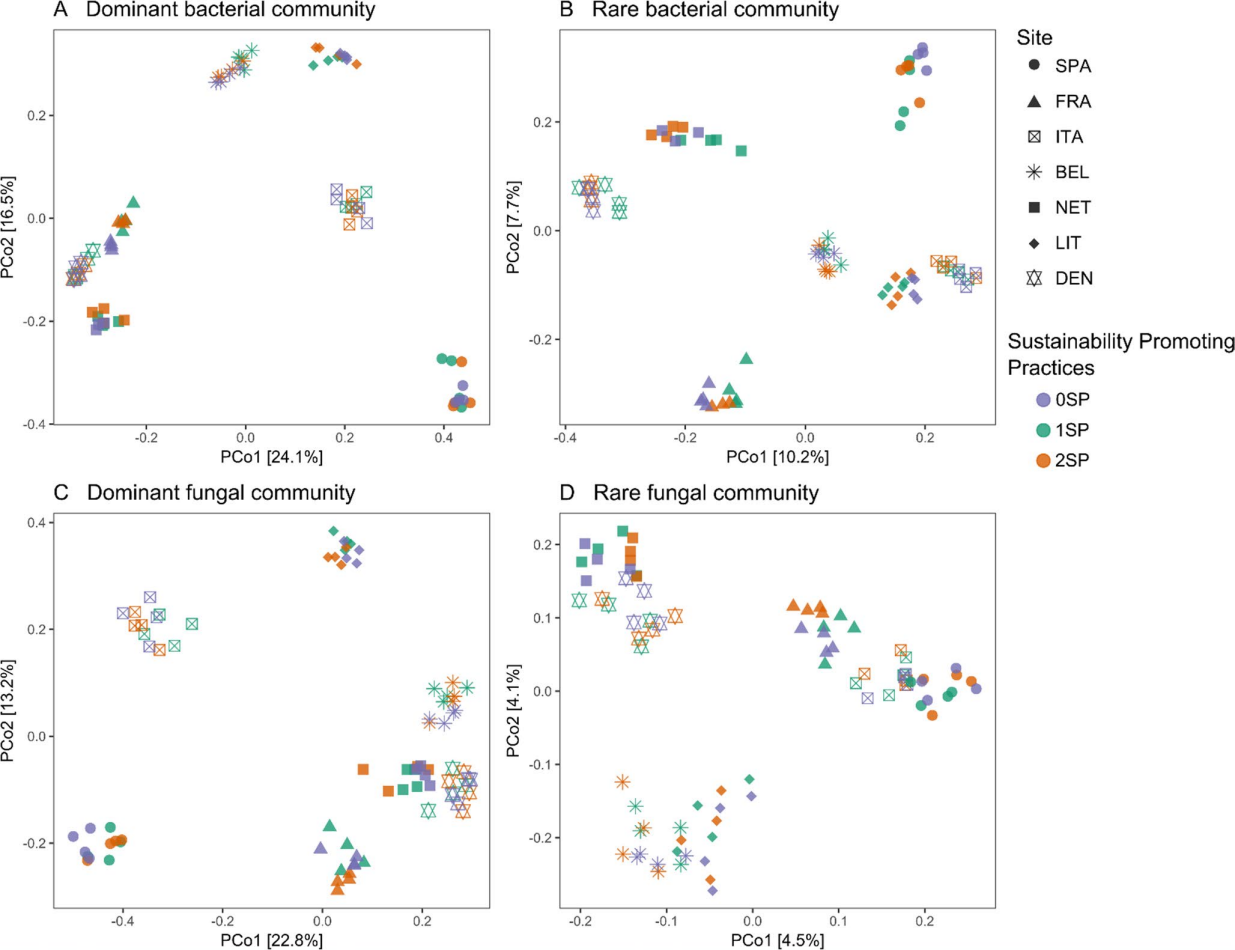
Principal Coordinate Analysis (PCoA) based on the Bray-Curtis dissimilarity matrix of sub-communities of soil bacteria (**A** – **B**) and soil fungi (**C** – **D**) across sites. Different shapes represent seven European countries. Colours represents the three sustainability-promoting practices

The compositions of the soil microbial communities (bacterial and fungal sub-communities) in the different core sites were clustered along the PCA1 (24.1%, 10.2%, 22.8%, and 4.5% of explained variance, respectively) following a geographic gradient from northern sites such as DEN/NET to southern sites such as SPA/ITA (Fig. 2). Although the proportion of variance explained by the first axis was relatively low for each sub-community, the two-way PERMANOVA tests revealed that core sites, treatments, and their interactions had significant effects on most bacterial and fungal sub-community compositions (Table S1).

The number of sustainability-promoting practices applied showed significant shifts in the beta diversity of the microbial sub-communities across the majority of core sites, according to one-way PERMANOVA analysis (Table S2) except for the DEN core site, where the treatments did not affect microbial structure in any sub-community.

In the dominant bacterial sub-community, the bacterial composition shifted by the number of sustainability-promoting practices applied in four core sites. In FRA and LIT, both 1SP and 2SP showed a similar shift compared to the 0SP, whereas in BEL and ITA, only the 1SP, but not the 2SP treatment shifted the community composition (Fig. S2 A). A similar trend was detected in the rare bacterial sub-community those core sites, but additionally, the NET core site also seemed to show a shift in the community composition, caused by the 1SP (Fig. S2B).

For the dominant fungal sub-community, significant compositional shifts were detected under the 1SP or 2SP treatments in all core sites except DEN. In FRA and LIT core sites, both 1SP and 2SP treatments shifted the composition similarly compared to the 0SP, without differences among them. In SPA and NET, only the higher intensification (2SP) shifted the dominant fungal composition compared to the 1SP, whereas in the ITA core site, only the 1SP caused changes in the dominant fungal structure (Fig. S2C). For the rare fungal sub-community, only SPA, BEL, and LIT showed statistical differences in the community structure, where both 1SP and 2SP shifted the composition similarly, compared to 0SP (Fig. S2 D).

**Fig. 3:**
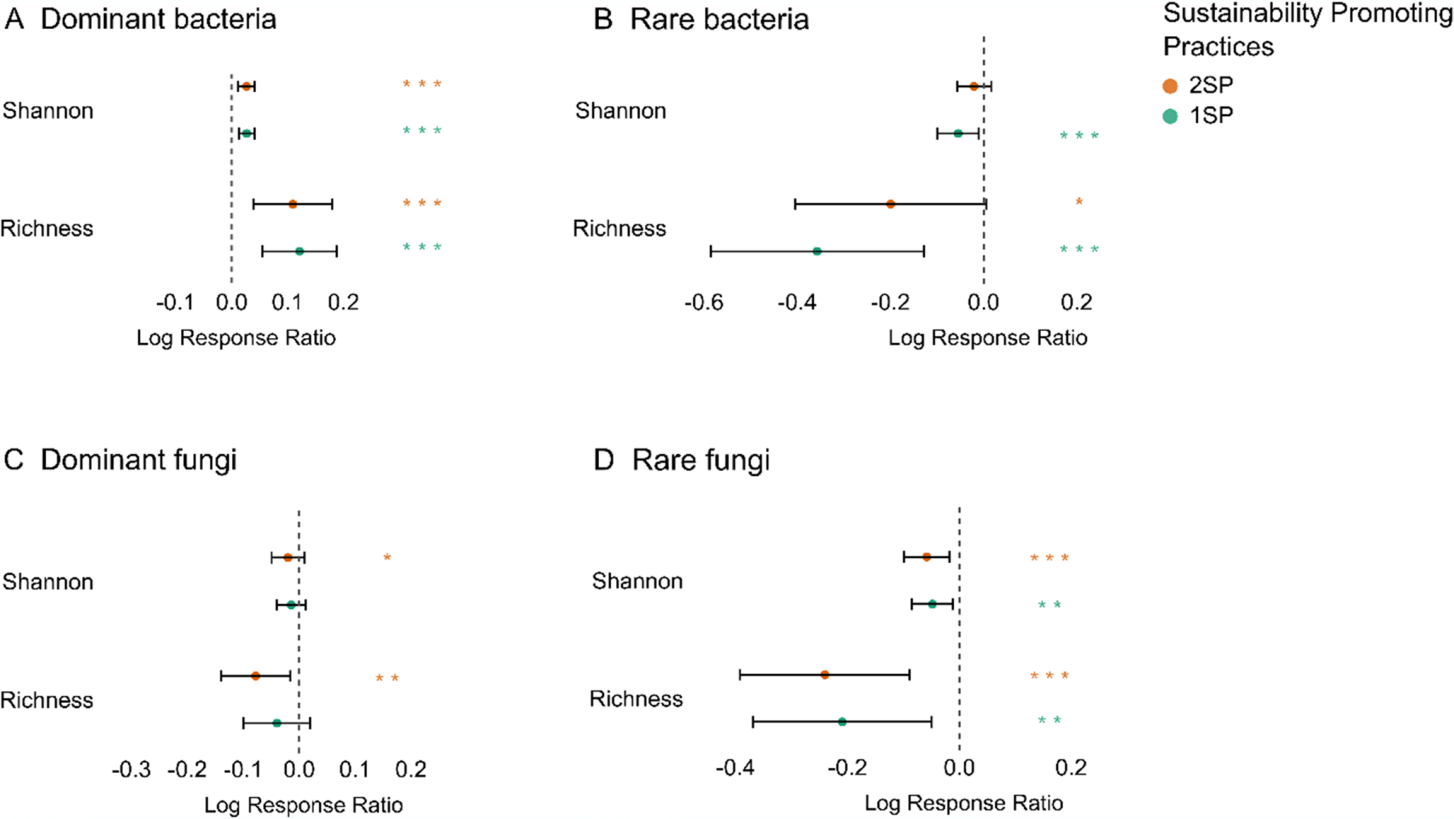
Mean Log Ratio Response (LRR) values of alpha diversity indexes and their 95% confidence intervals for dominant and rare bacteria (**A**, **B**) and dominant and rare fungi (**C**, **D**). Asterisks (*) indicates a significant effect (* *p* < 0.05, ** *p* < 0.01, *** *p* < 0.001) according to a linear mixed models using the site as random factor

The size effect (LRR) of the alpha bacterial diversity indexes increased significantly in the dominant bacteria sub-community with 1SP and 2SP, whereas for rare bacterial taxa, the diversity indexes decreased significantly in both cases, specially, with 1SP (Fig. 3A - B). For dominant and rare fungi, the diversity indexes decreased in both treatments (1SP and 2SP) (Fig. 3C-D).

### Effect of Sustainability-Promoting Practices and Bioclimatic and Soil Properties on the Sub-Communities of Soil Bacterial and Fungal Communities

To link individual soil properties and sub-communities’ dissimilarity, we performed partial Mantel tests controlling for geographical distance (Table 2). Total and organic carbon, total nitrogen, ammonium, and available phosphorus showed consistently significant relationships (*p* ≤ 0.01) with all subcommunities, whereas nitrate exhibited weaker and less consistent correlations. When considering the combined matrix of soil variables, the correlations remained significant, particularly for fungal sub-communities.

**Table 2:** Partial mantel tests of microbial sub-communities Bray – Curtis dissimilarities and environmental variables controlling for geographical distance

Variables	Dominant bacteria		Rare bacteria		Dominant fungi		Rare fungi	
	*r*	*p*	*r*	*p*	*r*	*p*	*r*	*p*
Total C	0.28	0.001	0.26	0.001	0.37	0.001	0.36	0.001
Organic C	0.30	0.001	0.26	0.001	0.37	0.001	0.38	0.001
Total N	0.31	0.001	0.24	0.001	0.41	0.001	0.37	0.001
NO_3_	0.07	0.016	0.04	0.081	0.15	0.007	0.13	0.001
NH^4^	0.14	0.002	0.11	0.001	0.24	0.001	0.24	0.001
P available	0.15	0.001	0.09	0.002	0.22	0.001	0.24	0.001
Variables distance matrix	0.31	0.001	0.24	0.001	0.44	0.001	0.42	0.001

Principal coordinate analysis 1 (PCoA1) of beta diversity and microbial sub-communities showed a significant correlation with latitude and bioclimatic conditions (Table S3, Fig. S4 A-C). The relationship between the microbial sub-communities and latitude showed a stronger response in the fungal sub-communities than in the bacterial sub-communities (Table S3, Fig. S4 C). However, only the rare fungal community was significantly influenced by longitude (Table S3, Fig. S4 B).

The bioclimatic conditions were different across the core sites. Core sites NET, BEL, and DEN were grouped according to high precipitations, whereas ITA, FRA, and SPA correlated with high temperatures (Fig. S3 A). The first component of the principal component analysis (PCA1) strongly correlated with the geographical coordinates, especially with latitude (Fig. S5). The soil properties were grouped according to core site. Three groups were observed: ITA correlated with the highest content of NO_3_^-^ and NH_4_^+^; FRA highly correlated with the highest values of total organic carbon; and a third group (SPA, BEL, LIT, NET, and DEN) correlated negatively with total organic carbon, total carbon, and total nitrogen (Fig. S3 B).

### Effect of Sustainability-Promoting Practices on the Sub-Communities of Soil Bacteria and Fungi Communities

**Fig. 4:**
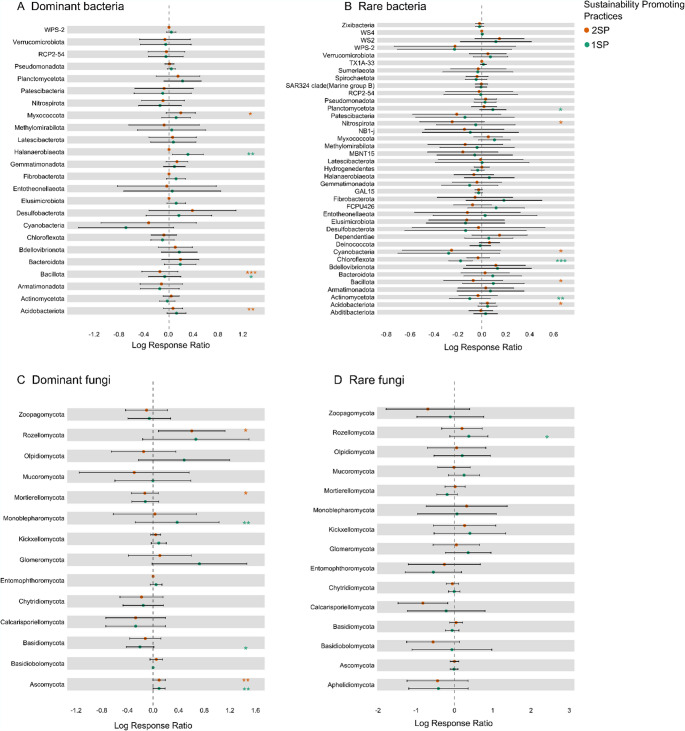
Forest plot showing the Mean Log Ratio Response (LRR) values of bacterial and fungal phyla and their 95% confidence intervals in the dominant bacterial community (A), rare bacterial sub-community (B), dominant fungal sub-community (C) and rare fungal sub-community (D) under different sustainability-promoting practices. Asterisks (*) indicates a significant effect (* p < 0.05, ** p < 0.01, *** p < 0.001) according to a linear mixed models using the site as random factor

The forest plot (Fig. 4) showed the log response ratio (LRR) of microbial sub-communities with 1SP and 2SP compared to 0SP. The number of phyla that decreases or increases in each subcommunity is summarized in the table S5. The LRR of dominant bacterial phyla (24 taxa) across all the core sites showed that two phyla increased significantly with 2SP (Acidobacteriota and Myxococcota) whereas Halanaerobiaeota increased only with 1SP. Additionally, the filum Bacillota decreased significantly in both treatments, especially in the 2SP (Fig. 4A). For rare bacteria, we found more phyla (39 taxa) but only Acidobacteriota increased significantly across core sites with 2SP, whereas the phyla Bacillota, Cyanobacteria and Nitrospirota decreased. Similarly, with 1SP, only the phylum Planctomycetota increased significantly, whereas the Actinomycetota and Chloroflexota phyla decreased (Fig. 4B).

For the dominant fungi (14 taxa), Ascomycota increased significantly with 1SP and 2SP. We observed a significant increase of Rozellomycota and a decrease of Mortierellomycota with 2SP, whereas under 1SP, we observed an increase of Monoblepharomycota and a decrease of Basidiomycota (Fig. 4C). Interestingly, in the rare fungal sub-community, only Rozellomycota showed a significant increase with 1SP.

**Fig. 5:**
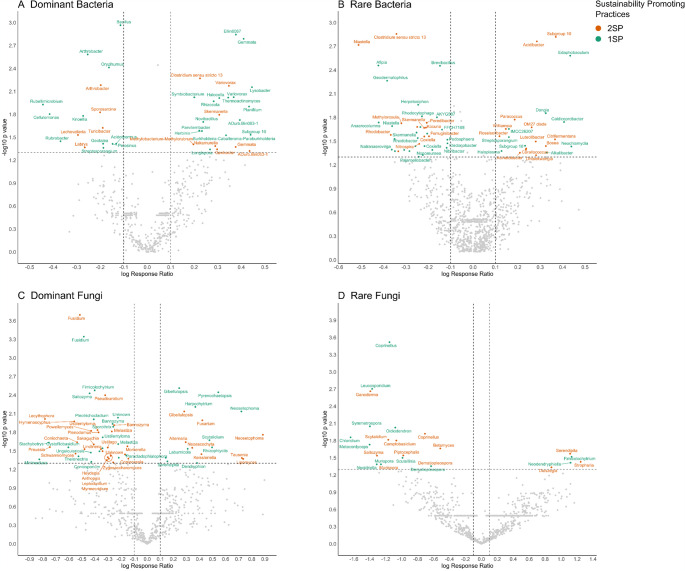
Volcano plot showing the mean Log Ratio Response (LRR) values of bacterial and fungal genus that were significantly enriched or reduced in the dominant bacterial sub-community (**A**), rare bacterial sub-community (**B**), dominant fungal sub-community (**C**) and rare fungal sub-community (**D**) under different sustainability-promoting practices. Vertical dashed lines indicate the 0.1 change threshold. The horizontal dashed lines represent the p values below 0.05 according to the linear mixed models, using the site as random factor

The volcano plot shows the LRR of the genera that significantly increased or decreased in the different sub-communities of bacteria and fungi with 1SP and 2SP compared to 0SP (Fig. 5). In Table S5 are summarized the number of taxa which decreases or increases in each sub-community. Core sites with 2SP, showed that nine of the dominant bacterial genera increased over the 0.1% threshold compared to the 0SP including *Gaiella*, *Geobacter* and *Gemmata*, whereas with the 1SP, 17 genera increased significantly over the threshold, including *Lysobacter*, *Variovorax*, *Rhizocola* or *Gaiella*. Contrary, 12 genera decreased significantly (Fig. 5A). In the rare bacterial sub-community, 13 genera increased significantly over the threshold (0.1%) under 2SP compared to 0SP, including *Bosea*, *Acidibacter*, *Coxiella* or *Nitrospira*, whereas with 1SP, 9 genera increased significantly like *Dongia*, *Alkalibacter* or *Caldicoprobacter* (Fig. 5B).

In the dominant fungi sub-community, 12 genera increased over the threshold (0.1%) with 2SP like *Fusarium*, *Tausonia*, *Gibellulopsis*, *Paraphoma*, *Neosetophoma* or *Lipomyces*. Similarly, with 1SP, also 12 fungal genera increased in the dominant fungi sub-community, including *Neosetophoma*, *Gibellulopsis* or *Laburnicola* (Fig. 5C). In the rare fungi sub-community 3 taxa increased over the threshold (0.1%) significantly with 2SP (*Stropharia*, *Serendipita* and *Dioszegia*), and with 1SP, only 2 taxa increased, and two genera increased significantly (*Fimicolochytrium* and *Neodendryphiella*) (Fig. 5D).

## Discussion

The study of soil microbial sub-communities is essential for soil quality preservation and the optimization of soil management, since soil microbes are sensitive to environmental impact and are one of the best predictors of soil quality [70]. Despite that in our study we did not directly measure microbial functions, and focused on a taxonomic approach, this can provide valuable insights into soil functionality, as shifts in the abundance of particular microbial groups are often linked to changes in key ecological processes [42, 43]. However, it is important to note that taxonomic data alone cannot be used to infer microbial functions unequivocally, as the same taxa may play different roles depending on environmental context, management conditions or geographical distribution [71]. Additionally, analyzing dominant and rare taxa sub-communities separately is essential for understanding the process of soil ecosystems in different study sites or agriculture practices, allowing to capture the full diversity of the soil microbiome, as rare taxa are often overlocked or masked by dominant taxa in standard analyses [30, 31, 72]. Whereas dominant microorganisms might be the major contributors to ecosystem functioning and maintaining the microbial communities structure [32], several studies have also highlighted that the rare microbial sub-community is key for regulating ecosystem functions, displaying a disproportional role in biochemical cycles compared to dominant species, providing complementary functions to support ecosystems stability, and are more easily affected by environmental disturbances [39, 44, 72].

Our results indicate that both dominant and rare microbial sub-communities respond to sustainability-promoting agricultural practices differently, with dominant taxa generally showing stronger shifts in accordance to other researchers [31, 33], while site-specific environmental drivers played a differential role across sub-communities, with latitude and climate affecting all sub-communities similarly, longitude affecting only the rare fungal fraction, and soil properties showing a significant relationship with all the sub-communities, particularly with the fungal communities.

### Dominant and Rare Microbial Communities in Different European Core Sites

Although rare microbial taxa have been often overlooked in analyses [73], they are known as a source of genetic resources and to become particularly relevant under environmental shifts, such as soil degradation, by adapting to new conditions and potentially replacing previously dominant taxa, contributing to ecosystem stability [30, 74, 75]. In our study, the rare bacterial sub-community consistently showed higher richness than that of the dominant sub-communities across European core sites as expected, which is in accordance to previous studies [72], supporting the idea that the rare sub-community is the main component of microbial diversity and could indicate higher resilience [76]. By contrast, in the fungal community, the dominant taxa exhibited slightly higher richness than the rare ones, suggesting that fungal assemblages may follow different diversity–stability dynamics than bacterial communities [77–79].

### Diversity Indexes Related to the Number of Sustainability-Promoting Practices

The bacterial and fungal sub-communities differed according to the latitude and bioclimatic conditions of the core sites, indicating a gradient from the core sites in the north of Europe to the south. This is in line with Banerjee et al. [77], who demonstrated that the relationships of the microbial communities could be explained by geographic proximity, considering climatic conditions and edaphic properties [74, 80].

The alpha-diversity indexes only increased significantly in the dominant bacteria sub-community under both 1SP and 2SP, that could indicate that the dominant bacterial sub-community is sensitive to changes induced by sustainability-promoting agriculture practices. Nevertheless, previous studies have pointed out that alpha-diversity indices are generally less sensitive for differentiating microbial communities compared to other indexes [28, 47, 81]. In contrast, in both fungal sub-communities, alpha-diversity decreased, highlighting the fungal different response patterns to the implementation of sustainability-promoting practices [82].

The beta diversity showed that, across the different core sites, dominant fungi were more sensitive to agricultural practices than the rare fungal sub-community and both bacterial sub-communities. Significant shifts under 1SP or 2SP compared with 0SP were detected in six core sites for dominant fungi, whereas only four core sites showed changes in dominant bacteria, five in rare bacteria, and three in rare fungi. This indicates that dominant fungi exhibited the highest number of significant responses to sustainability-promoting practices across sites. However, this is contrary to Banerjee et al. [77], who showed rare fungi to be more sensitive to disturbances. The principal differences among the sub-communities of bacteria and fungi were observed with 2SP, with the exception of the BEL core site, where 1SP, involving the addition of cattle manure, shifted the microbial communities greatly which agrees with Semenov et al. [83]. Our results indicated that, in general, increasing the number of sustainability-promoting practices tended to strengthen the effects on the microbial community, most notably in the dominant fungal community, followed by the bacterial rare, and to a lesser extent the bacterial dominant sub-communities respectively. However, the effects varied across core sites, reflecting differences in applied treatments and site-specific edaphic and climatic conditions.

### Shift of Bacterial and Fungal Sub-Communities with the Number of Sustainability-Promoting Practices

Identifying the microbial taxa responsible for shifts in the communities with different agronomical practices is one of the most important factors in the study of the microbiome, as these taxa participate in nearly all soil biological processes, however, the use of amplicon sequencing only shows potential functionality. In the dominant bacterial sub-communities, phyla such as Acidobacteriota and Myxococcota have increased under the 2SP where compost addition, residue retention and no-till farming practices were applied [14, 84, 85]. Notably, Acidobacteriota community contribute to essential soil processes, including various roles in the nitrogen, phosphorous, sulphur and carbon cycles [85] whereas Myxococcota members are important micropredators, that can play an essential role shaping microbial communities in agricultural soils [14, 86]. Unexpectedly, phyla like Bacillota, previously known as Firmicutes, which members are related to important roles in agroecology [87] decreased with both 1SP and 2SP across core sites, contrary to other studies results [88, 89].

Some dominant bacterial taxa were identified as increasing with both treatments across the core sites including species of *Variovorax* (Pseudomonadota), known as endophytes that can promote plant growth in poor soils [90]. Microorganisms of the genus *Geobacter* participates in carbon and nitrogen cycling in soils, contributing to CO_2_ fixation in soils [91] increased significantly with 2SP. Whereas microorganisms of the genus *Lysobacter* (Pseudomonadota), known for its capacity to suppress pathogens, mineralize nutrients in the soil, and promote plant growth and yield [92] increased significantly only with 1SP.

Among the rare bacterial taxa, Acidobacteriota and Planctomycetota related to plant cover [93] increased greatly under 2SP and 1SP treatments respectively. According to the number of practices, different genera such as *Edaphobaculum* (Bacteroidota), associated to soil fertility [94] and *Caldicoprobacter* (Bacillota), associated to biodegradation of hemicelluloses in soils [95] increased significantly under the 1SP. While under 2SP, genera such as *Bosea*, considered a disease-suppressing bacteria [96], and *Paracoccus*, related to synthetic biodegradation of materials such as pesticides or plastics, along with organic compounds [74] increased in the fungal sub-communities. Interestingly, the phylum Glomeromycota (Arbuscular mycorrhizal fungi (AMF)) which is known as a root colonizer for most terrestrial plants since it facilitates mineral nutrient uptake from the soil in exchange for plant-assimilated carbon [97] increased greatly with 1SP. However, the AMF sequences might be underrepresented in this study since we used the ITS2 region instead of fungal-specific primers. This could lead to an underestimation of the diversity and abundance of these fungi [98, 99]. In contrast, with 2SP Rozellomycota (also known as Cryptomycota) increased, according to Olayemi et al. [100] and Muturi et al. [101] that reported the increase of Rozellomycota under different sustainability-promoting practices like cover crops, no-till farming, residue retention, and organic fertilizers. Whereas, Mortierellomycota which promotes plant growth through phytohormone production and phosphorous solubilization [102] decreased in both treatments (1SP and 2SP).

In the dominant fungi sub-community, genera such as *Neosetophoma* (Ascomycota), a litter saprotroph [103] and *Gibellulopsis* (Ascomycota) a biocontrol agent that can act as a plant endophyte protecting crops against *Verticillum* [75, 104] increased significantly under both sustainability-promoting practices (1SP and 2SP), whereas *Pyrenochaetopsis* (Ascomycota), identified as an important indicator of plant agronomical traits ([105] only increased under 1SP practices. Under the 2SP practices, the *Neosetophoma* genus (Ascomycota), related to litter decomposition and no-till farming [103, 106] increased significantly across the core sites. Interestingly, we also observed an increase of *Fusarium* under 2SP practices. Although some management strategies, such as reduced tillage or the application of green manure, can create conditions (e.g., higher organic matter and moisture) that favor *Fusarium* proliferation, considering the existence of pathogenic and non-pathogenic [107–109]. Further analysis will be necessary to address it. Among rare fungi few taxa increased significantly across the core sites, interestingly in the 2SP practices, *Stropharia* (Basidiomycota) and *Serendipita* (Basidiomycota), an endophytic fungus with several applications in agriculture [110] increased significantly.

### Drivers of Microbial Communities

We also investigated the drivers of microbial community composition and structure across European agricultural soils. Large-scale surveys of microbial fungi typically focus on natural communities rather than agricultural systems [111, 112]. Thus, in this study, we assessed how the microbial communities were distributed across different European core sites. We observed that the structure of microbial sub-communities was primarily determined by the distance to the equator, with core sites clustering along a north-south axis, while little relationship was observed along the east-west axis. This pattern reflects the strong influence of latitude on bioclimatic conditions, as northern and southern Europe differ greatly on climatic conditions, mainly influenced by proximity to the Mediterranean Sea (south), which is characterized by higher temperatures, lower precipitation, and a summer drought period, in difference to the northern latitudes [113]. This relationship was more pronounced in the fungal communities than in the bacterial ones, consistent with previous studies observing that bacterial communities separated geographically tend to cluster better according to soil properties than geographical proximity [114, 115].

Our results revealed significant relationships between microbial communities and soil properties, with stronger association observed for the fungal communities. Moreover, fungal communities were also more sensitive to bioclimatic gradients, particularly the rare fungal sub-community. These findings indicate that fungal communities exhibit more pronounced changes than bacterial communities across the core sites studied. These results are in accordance with previous studies where fungal communities, particularly the rare ones were found to be more sensitive to bioclimatic conditions such as precipitation [78, 116, 117]. Bacterial communities, despite significant, show weaker responses to both climatic and edaphic gradients [23, 112, 118]. Altogether, these results reinforce the idea that fungal assemblages are more environmentally responsive than bacterial ones at large spatial scales.

## Conclusion

Our findings demonstrates that the response of soil microbial sub-communities to sustainability-promoting agricultural practices is highly variable across different European core sites, strongly driven by bioclimatic conditions and agricultural practices. Overall, bacterial richness was higher in the rare sub-community, but unexpectedly fungal richness was slightly greater in the dominant sub-community. Beta diversity analyses revealed that dominant fungi as the most responsive group to sustainability-promoting practices, exhibiting significant shifts across a larger number of core sites compared to other microbial sub-communities. Moreover, sustainability-promoting practices promoted microbial groups involved in carbon and nutrient cycling, plant growth promotion, and pathogen suppression, particularly within members of Acidobacteriota, Bacillota, Myxococcota, Glomeromycota, and Rozellomycota.

## Supplementary Information

Below is the link to the electronic supplementary material.

Supplementary Material 1 (DOCX 13.9 MB)

## Data Availability

Sequencing data (16S and ITS rRNA gene amplicon) were deposited at the Sequence Read Archive https://www.ncbi.nlm.nih.gov/sra under the BioProject accession PRJNA1380092. Data will be made available on request.
