# Supplementary material for: The Influence of Agroecological Intensification on Dominant and Rare Microbial Communities Across Diverse European Countries
Source: Microb Ecol. 2025 Dec 19;89(1):29. doi: 10.1007/s00248-025-02655-5 (PMC12808173; doi:10.1007/s00248-025-02655-5)
Supplement: Supplementary file 1 — Supplementary Material 1 (DOCX 13.9 MB) [file 248_2025_2655_MOESM1_ESM.docx]

| **Table S1.** Two-way permanova results calculated with 999 permutations of Bray - Curtis dissimilarity distance matrix for microbial sub-communities. | | | | | |
| --- | --- | --- | --- | --- | --- |
| **Sub-community** | **Factor** | **R2** | **F** | **Pr(>F)** | **Significance** |
| Dominant Bacteria | Treatment | 0.01 | 1.57 | 0.078 | ns |
|  | Site | 0.79 | 49.93 | 0.001 | ** |
|  | Treatment:Site | 0.04 | 1.37 | 0.016 | * |
| Rare Bacteria | Treatment | 0.02 | 1.23 | 0.075 | ns |
|  | Site | 0.43 | 9.51 | 0.001 | ** |
|  | Treatment:Site | 0.11 | 1.19 | 0.008 | ** |
| Dominant Fungi | Treatment | 0.01 | 1.91 | 0.009 | ** |
|  | Site | 0.69 | 32.77 | 0.001 | ** |
|  | Treatment:Site | 0.07 | 1.76 | 0.001 | ** |
| Rare Fungi | Treatment | 0.02 | 1.10 | 0.079 | ns |
|  | Site | 0.21 | 3.50 | 0.001 | ** |
|  | Treatment:Site | 0.13 | 1.09 | 0.006 | ** |

| **Table S2.** Permanova results calculated with 999 permutations of Bray - Curtis dissimilarity distance matrix for each different core site. | | | | |
| --- | --- | --- | --- | --- |
| **Dominant Bacteria** | **R2** | **F** | **Pr(>F)** | **Significance** |
| Spain | 0.223 | 1.288 | 0.224 | ns |
| France | 0.307 | 1.775 | 0.005 | ** |
| Italy | 0.233 | 1.365 | 0.008 | ** |
| Belgium | 0.296 | 1.892 | 0.001 | ** |
| Netherlands | 0.223 | 1.145 | 0.134 | ns |
| Lithuania | 0.267 | 1.636 | 0.040 | * |
| Denmark | 0.190 | 0.941 | 0.678 | ns |
| **Rare Bacteria** | **R2** | **F** | **Pr(>F)** |  |
| Spain | 0.206 | 1.168 | 0.126 | ns |
| France | 0.257 | 1.385 | 0.006 | ** |
| Italy | 0.206 | 1.168 | 0.024 | * |
| Belgum | 0.210 | 1.198 | 0.005 | ** |
| Netherlands | 0.223 | 1.150 | 0.012 | * |
| Lithuania | 0.229 | 1.339 | 0.019 | * |
| Denmark | 0.202 | 1.013 | 0.413 | ns |
| **Dominant Fungi** | **R2** | **F** | **Pr(>F)** |  |
| Spain | 0.243 | 1.442 | 0.028 | * |
| France | 0.377 | 2.728 | 0.005 | ** |
| Italy | 0.298 | 1.908 | 0.006 | ** |
| Belgium | 0.252 | 1.515 | 0.007 | ** |
| Netherlands | 0.239 | 1.413 | 0.019 | * |
| Lithuania | 0.369 | 2.627 | 0.001 | ** |
| Denmark | 0.160 | 0.855 | 0.798 | ns |
| **Rare Fungi** | **R2** | **F** | **Pr(>F)** |  |
| Spain | 0.200 | 1.126 | 0.046 | * |
| France | 0.200 | 1.126 | 0.065 | ns |
| Italy | 0.182 | 1.004 | 0.482 | ns |
| Belgium | 0.205 | 1.161 | 0.035 | * |
| Netherlands | 0.185 | 1.024 | 0.379 | ns |
| Lithuania | 0.217 | 1.244 | 0.003 | ** |
| Denmark | 0.179 | 0.980 | 0.592 | ns |

| **Table S3.**  Spearman correlation test of the first principal coordinates analysis (PCoA1) of the different sub-communities and the geographical location (Latitude and longitude) and the first component of the principal component analysis (PCA1) of the bioclimatic conditions. | | | |
| --- | --- | --- | --- |
| **Variable** | **Sub-community** | **r** | **Significance** |
| Latitude | Dominant Bacteria | -0.71 | *** |
|  | Rare Bacteria | -0.64 | *** |
|  | Dominant Fungi | 0.754 | *** |
|  | Rare Fungi | -0.80 | *** |
| Longitude | Dominant Bacteria | -0.09 | ns |
|  | Rare Bacteria | 0.16 | ns |
|  | Dominant Fungi | 0.16 | ns |
|  | Rare Fungi | -0.27 | * |
| PCA1 bioclimatic conditions | Dominant Bacteria | 0.60 | *** |
|  | Rare Bacteria | 0.63 | *** |
|  | Dominant Fungi | -0.75 | *** |
|  | Rare Fungi | 0.71 | *** |

| **Table S4**. Number of taxa decreasing / increasing significantly in each subcommunity according to linear mixed models using the site as random factor. | | | | |
| --- | --- | --- | --- | --- |
| **Sub - Community** | **Phyla** | | **Genera** | |
|  | **1SP** | **2SP** | **1SP** | **2SP** |
| Dominant Bacteria | 1 / 1 | 1 / 2 | 11 / 17 | 5 / 8 |
| Rare Bacteria | 2 / 1 | 3 / 1 | 26 / 9 | 12 / 13 |
| Dominant Fungi | 1 / 2 | 0 / 3 | 19 / 10 | 26 / 8 |
| Rare Fungi | 0 / 1 | 0 / 0 | 9 / 2 | 7 / 3 |


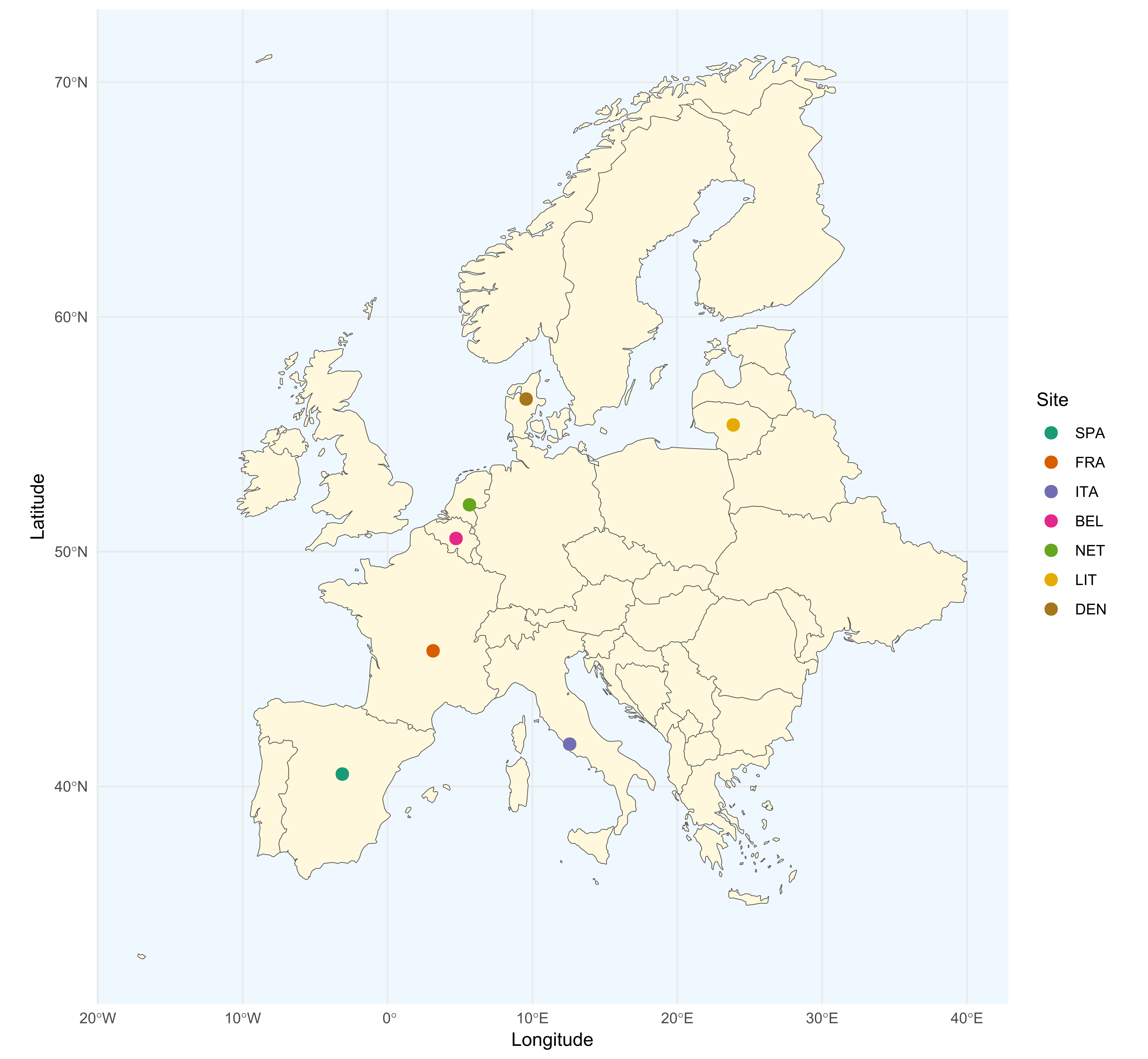


**Figure S1**: Geographic situation of the different core sites in the EJP Soil project (AGROecoSeq).


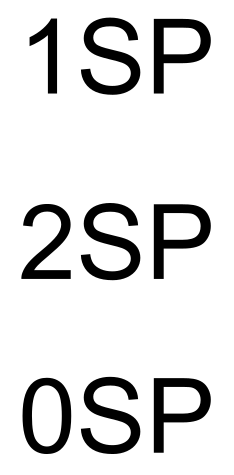

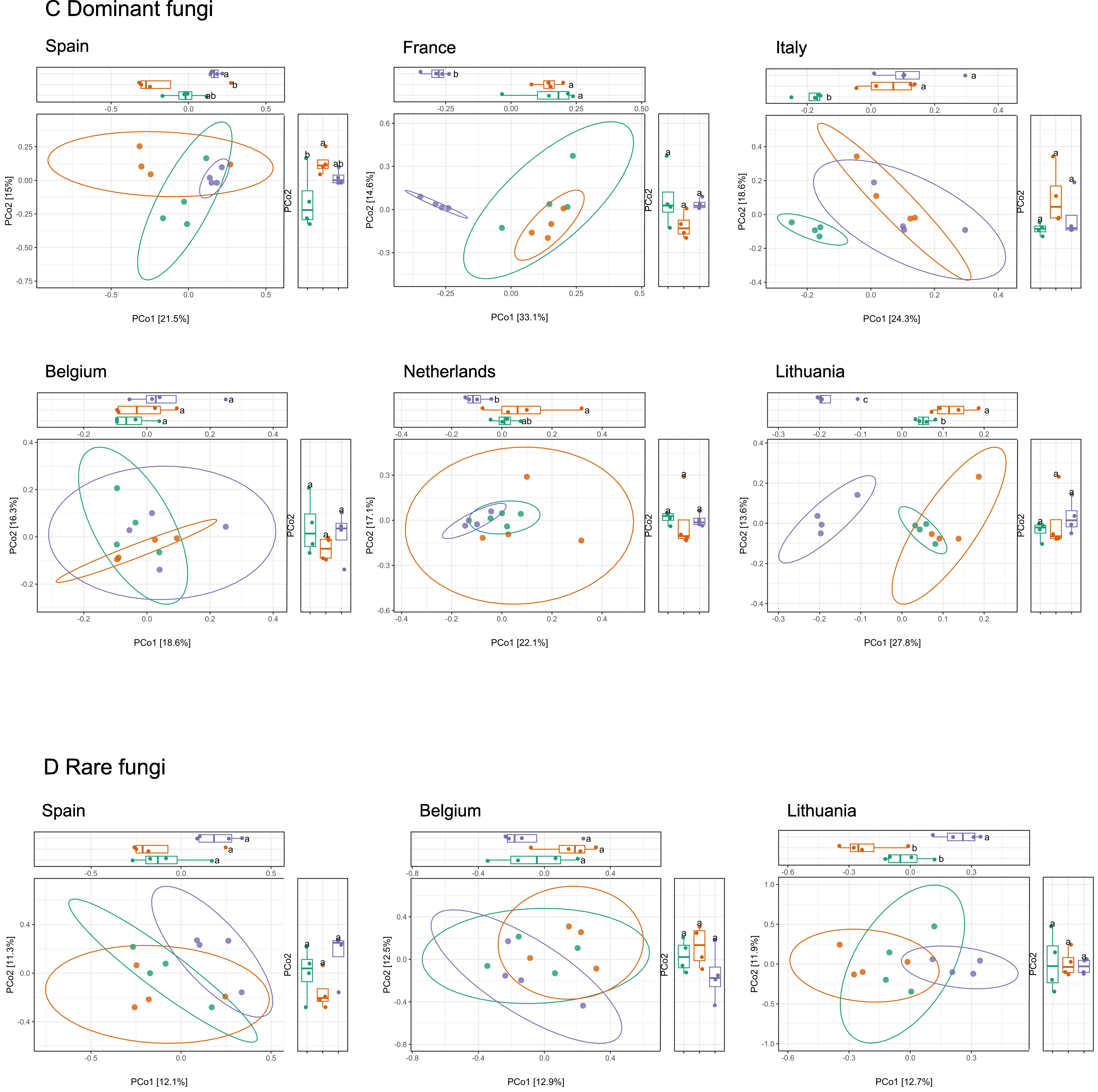





**Figure S2:** Principal Coordinate Analysis (PCoA) showing the Bray-Curtis dissimilarity matrix of dominant and rare soil bacteria (A – B) and dominant and rare soil fungi (C – D) across countries with significant differences according to PERMANOVA analysis. Boxplots shows differences between treatments in the PCoA components 1 and 2 according to Duncan´s Multiple Range post hoc test.


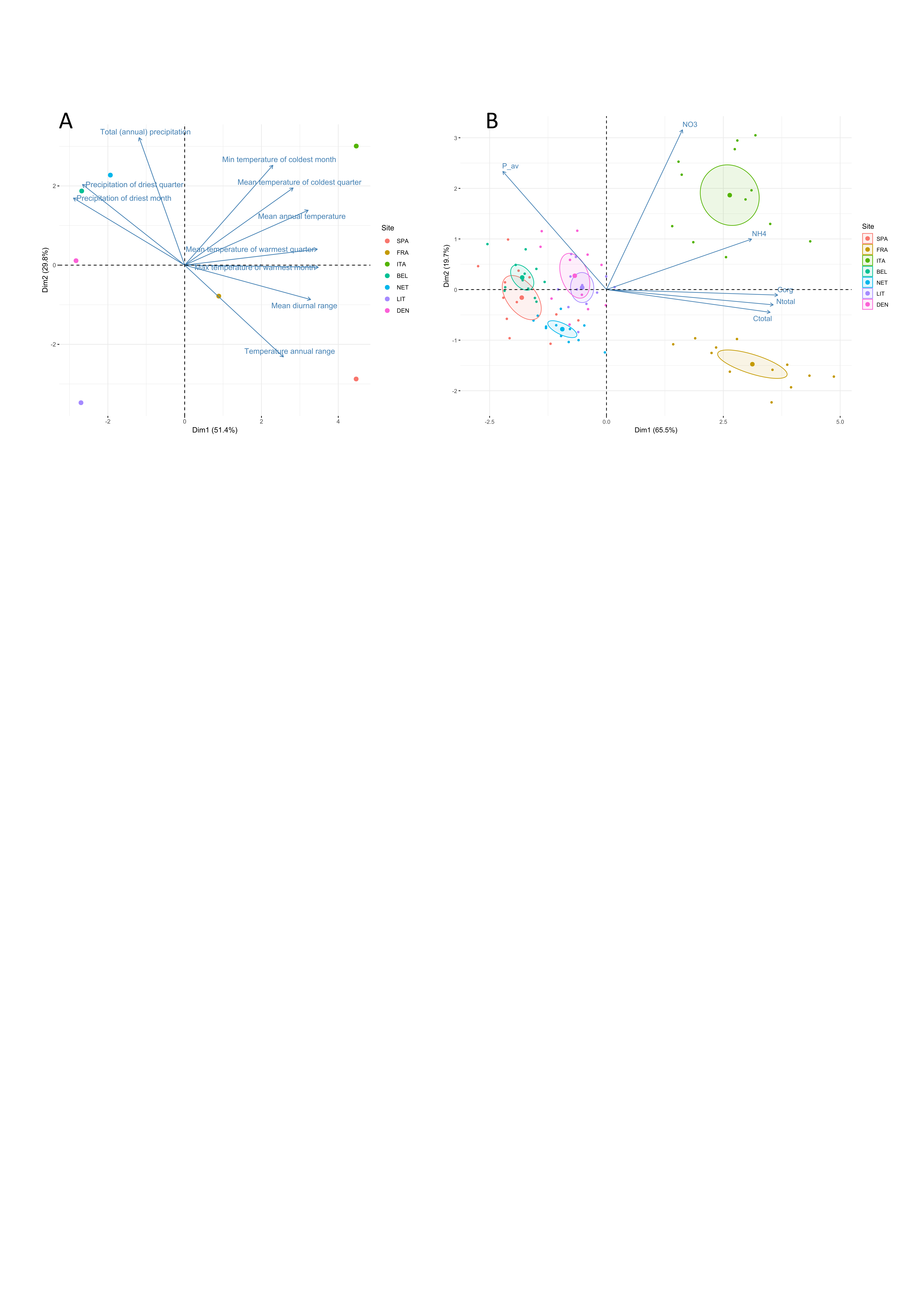


**Figure S3**: Principal component analysis (PCA) of bioclimatic variables obtained from the WorldClim database (A) and the soil properties (B)


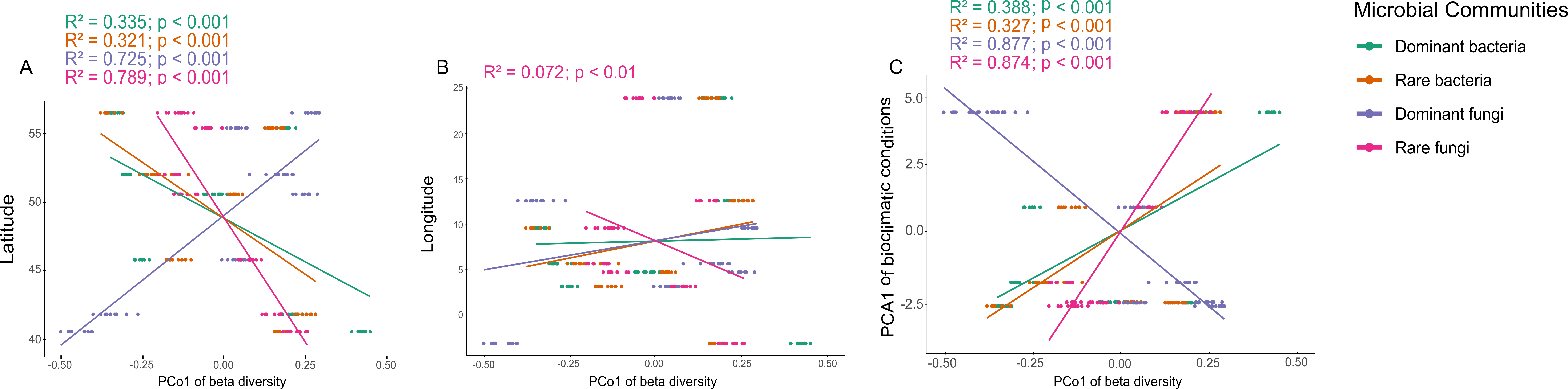


**Figure S4.** Linear regression between the different microbial community’s composition (principal coordinates analysis 1; PCoA1) and different variables: latitude gradient (A), longitude gradient (B), and the principal component 1 (PC1) of climatic conditions (C) determined by linear regression analysis. The lines show the linear regression and the adjusted R2 and p values indicate statistical significance.

B

A


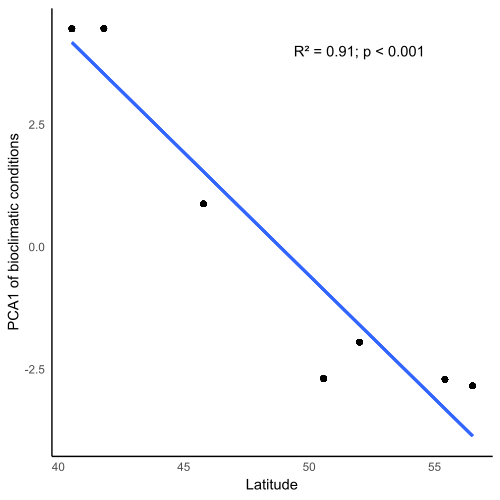

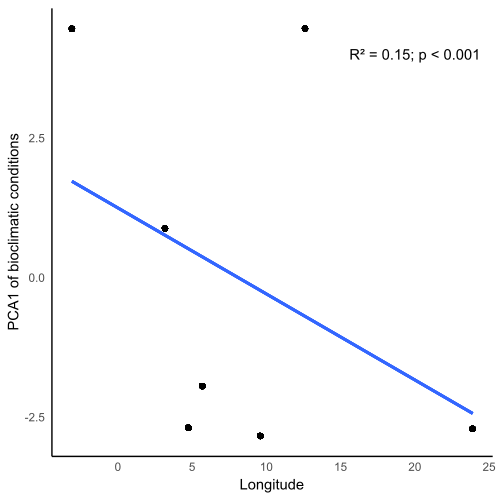


**Figure S5**: Relationship between the latitude (A) and longitude (B) with the first component (PCA1) of bioclimatic variables
